# Gray matter volume and estimated brain age gap are not linked with sleep‐disordered breathing

**DOI:** 10.1002/hbm.24995

**Published:** 2020-04-02

**Authors:** Bahram Mohajer, Nooshin Abbasi, Esmaeil Mohammadi, Habibolah Khazaie, Ricardo S. Osorio, Ivana Rosenzweig, Claudia R. Eickhoff, Mojtaba Zarei, Masoud Tahmasian, Simon B. Eickhoff, for the Alzheimer's Disease Neuroimaging Initiative

**Affiliations:** ^1^ Institute of Medical Science and Technology, Shahid Beheshti University Tehran Iran; ^2^ Non‐Communicable Diseases Research Center Endocrinology and Metabolism Population Sciences Institute, Tehran University of Medical Sciences Tehran Iran; ^3^ McConnell Brain Imaging Centre Montreal Neurological Institute, McGill University Montreal Quebec Canada; ^4^ Sleep Disorders Research Center Kermanshah University of Medical Sciences Kermanshah Iran; ^5^ Department of Psychiatry, Center for Brain Health, NYU Langone Medical Center New York New York USA; ^6^ Nathan S. Kline Institute for Psychiatric Research New York New York USA; ^7^ Sleep Disorders Centre Guy's and St Thomas' Hospital, GSTT NHS London UK; ^8^ Sleep and Brain Plasticity Centre, Department of Neuroimaging IOPPN, King's College London London UK; ^9^ Institute of Neuroscience and Medicine (INM‐1; INM‐7), Research Center Jülich Jülich Germany; ^10^ Institute of Clinical Neuroscience and Medical Psychology, Heinrich Heine University Düsseldorf Germany; ^11^ Institute of Systems Neuroscience, Medical Faculty, Heinrich‐Heine University Düsseldorf Germany

**Keywords:** age prediction, Alzheimer's disease, Alzheimer's Disease Neuroimaging Initiative, gray matter, mild cognitive impairment, sleep‐disordered breathing

## Abstract

Alzheimer's disease (AD) and sleep‐disordered breathing (SDB) are prevalent conditions with a rising burden. It is suggested that SDB may contribute to cognitive decline and advanced aging. Here, we assessed the link between self‐reported SDB and gray matter volume in patients with AD, mild cognitive impairment (MCI) and healthy controls (HCs). We further investigated whether SDB was associated with advanced brain aging. We included a total of 330 participants, divided based on self‐reported history of SDB, and matched across diagnoses for age, sex and presence of the Apolipoprotein E4 allele, from the Alzheimer's Disease Neuroimaging Initiative (ADNI). Gray‐matter volume was measured using voxel‐wise morphometry and group differences in terms of SDB, cognitive status, and their interaction were assessed. Further, using an age‐prediction model fitted on gray‐matter data of external datasets, we predicted study participants' age from their structural images. Cognitive decline and advanced age were associated with lower gray matter volume in various regions, particularly in the bilateral temporal lobes. Brains age was well predicted from the morphological data in HCs and, as expected, elevated in MCI and particularly in AD subjects. However, there was neither a significant difference between regional gray matter volume in any diagnostic group related to the SDB status, nor in SDB‐by‐cognitive status interaction. Moreover, we found no difference in estimated chronological age gap related to SDB, or by‐cognitive status interaction. Contrary to our hypothesis, we were not able to find a general or a diagnostic‐dependent association of SDB with either gray‐matter volumetric or brain aging.

## 1 INTRODUCTION

Dementia syndromes including Alzheimer's disease (AD), are major global concerns, with a prevalence of 712 cases per 100,000 population in 2016, affecting 40–50 million people worldwide (Nichols et al., 2019). Considering that the number of AD patients has been more than doubled during the past three decades (Nichols et al., 2019), it is critical to unravel the predisposing risk factors (Xu et al., 2015). These include advanced aging of the world population, but also modifiable risk factors (Xu et al., 2015) such as diabetes (Vagelatos & Eslick, 2013), obesity (Alford, Patel, Perakakis, & Mantzoros, 2018), and sleep‐disordered breathing (SDB) (Emamian et al., 2016) Sleep‐disordered breathing ranges from partial (episodical) to complete airway obstruction leading to intermittent hypoxia, sleep fragmentation and intrathoracic pressure swings (Adult Obstructive Sleep Apnea Task Force of the American Academy of Sleep Medicine et al., 2009). A bidirectional relationship has been proposed between SDB, including its most common form (i.e., obstructive sleep apnea [OSA]), and AD. In particular, it has been suggested that patients with OSA are more likely to develop mild cognitive impairment (MCI) or dementia (Osorio et al., 2015; Yaffe et al., 2011). Moreover, our meta‐analysis demonstrated that the prevalence of OSA is five times higher in patients with AD than cognitively unimpaired individuals of the same age (Emamian et al., 2016).

Gray matter atrophy is a key feature of pathologic brain aging (Karas et al., 2004) and a common finding in the AD studies, starting primarily in the medial temporal region and then globally affecting the brain along the trajectory of disease (Fox & Schott, 2004; C. R. Jack et al., 2004; Pasquini et al., 2019). Morphometric analysis of the structural MRI images has shown to reliably reveal this effect (Good et al., 2001). While some studies have shown gray matter atrophy in brain regions like the hippocampus, a key region involved in AD, to be associated with SDB in non‐demented subjects (Joo et al., 2010; Joo, Jeon, Kim, Lee, & Hong, 2013; Morrell et al., 2010; Torelli et al., 2011; Weng et al., 2014), others have shown either null results (O'Donoghue et al., 2005; Yun et al., 2017) or paradoxical hypertrophy or thickening of gray matter in SDB (Baril et al., 2017; Kumar et al., 2014; Lin et al., 2016; Lundblad et al., 2014; Rosenzweig et al., 2013; Taylor et al., 2018) Discrepancy between these findings is attributed to variations in cognitive status of participants, definitions of SDB severity, and method of gray matter volume assessment (Baril et al., 2017; Sebastien Celle et al., 2016; Fatouleh et al., 2014; Joo et al., 2013; Kumar et al., 2014; Lin et al., 2016; Lundblad et al., 2014; Rosenzweig et al., 2013; Torelli et al., 2011). Thus, the contributing role of SDB in AD pathophysiology is still an open question.

Aside from regional atrophy in the medial temporal lobe, AD is associated with advanced multivariate patterns of brain aging. In particular, it has been demonstrated that individual subjects' age can be predicted from gray matter morphometry in the cognitively normal population using machine‐learning approaches (Varikuti et al., 2018). That is, models trained to predict individuals ages based on larger cohorts of reference images allow to estimate the age of a new person with a mean accuracy of 4–5 years (Franke, Luders, May, Wilke, & Gaser, 2012), while studies on neurodegenerative disorders showed a pattern of advanced aging, that is, a positive BrainAGE score (difference between the age predicted, based on the morphometric pattern, and chronological age) (Cole, Marioni, Harris, & Deary, 2019; Gaser et al., 2013; Löwe, Gaser, Franke, & Alzheimer's Disease Neuroimaging Initiative, 2016; Varikuti et al., 2018). Although it has been demonstrated that SDB was linked with an earlier age at cognitive decline and treatment of SDB postpones progression of cognitive impairment (Osorio et al., 2015), it remains unclear, whether and how SDB is associated with accelerated brain age and potential brain atrophy in AD.

The aim of the current study is to shed further light on the potential relationship between SDB and AD in terms of brain atrophy patterns at the regional and global levels, answering two questions. (a) Do patients with SDB show gray matter atrophy across or in interaction with cognitive status (healthy controls (HCs), MCI, and AD)? (b) Do patients with SDB show advanced brain aging across or in interaction with cognitive status? To this end, we used data from the Alzheimer's Disease Neuroimaging Initiative (ADNI), and established the validity of our methods by replicating previous findings for both aims in MCI and AD, and then assessed gray matter volume and BrainAGE differences between those patients with SDB compared to their counterparts, including interactions with cognitive status.

## 2 MATERIAL AND METHODS

### 2.1 Participants

Subjects were drawn from the ADNI database (adni.loni.usc.edu) (Petersen et al., 2010) based on their cognitive status and the medical history regarding SDB, as suggested previously (Osorio et al., 2015). Diagnoses of MCI and AD were based on the ADNI criteria. Subjects with self‐reported “sleep apnea” or “obstructive sleep apnea” (or “OSA”) symptoms or receiving treatment with “Continuous Positive Airway Pressure” (or “CPAP”) or “bilevel positive airway pressure” (or “BiPAP”/“BPAP”) were defined as “positive SDB.” Two independent physicians reviewed medical history to confirm diagnosis and grouping the subjects. Demographic and clinical variables were extracted for all individuals, missing covariate data were assessed and multiple imputation method including variables of sex, age, cognitive status, body‐mass index (BMI), and education years (Zhang, 2016) was used for five participants with missing data‐points on BMI (three subjects), and education years (two subjects). Using the 1:1 propensity score matching method, we assembled six distinct sub‐groups according to their cognitive (HC, MCI, AD) and SDB (positive or negative) status. Covariates included in the matching were age, sex, years of education, BMI, cognitive status (HC, MCI, AD), presence of the Apolipoprotein E4 (APOE4) allele, history of SDB treatment (only when matching between those with SDB subjects), T1 imaging protocol, and field strength (Table 1). To efficiently match the study diagnosis groups for age, we have included both baseline and follow‐up ADNI images in the propensity‐score matching model. Only subjects that passed the quality assessment of the CAT (Computational Anatomy Toolbox), including weighted image quality rating based on the basic image properties, noise and geometric distortions, as well as checking homogeneity through the sample, were included for our analyses.

**Table 1:** Characteristics of the study subjects

	Without sleep‐disordered breathing	With sleep‐disordered breathing	*p*‐Value
	*N*: 165	*N*: 165	
Age (mean [SD])	73.99 (7.70)	74.91 (7.18)	.26
Age range	56.1–91.9	58.1–91.2	—
Sex, female (%)	61 (37.0)	48 (29.1)	.16
Cognitive status (%)			1.00
Alzheimer's disease	24 (14.5)	24 (14.5)	
Mild cognitive impairment	111 (67.3)	111 (67.3)	
Healthy control	30 (18.2)	30 (18.2)	
Body‐mass index (mean [SD])	28.97 (5.95)	29.08 (5.45)	.86
Education years (mean [SD])	16.07 (2.75)	16.16 (2.65)	.74
Handedness, left (%)	18 (10.9)	18 (10.9)	1.00
APOE4 allele count (%)			.13
0	71 (46.7)	94 (58.0)	
1	64 (42.1)	53 (32.7)	
2	17 (11.2)	15 (9.3)	
MMSE (mean [SD])^a^	26.07 (4.13)	25.44 (4.93)	.25
CPAP/BiPAP/surgery (%)^a^	0 (0.0)	56 (33.9)	—
Protocol, MP‐RAGE (%)	118 (71.5)	124 (75.2)	.53

Abbreviations: BiPAP, bilevel positive airway pressure; CPAP, continuous positive airway pressure; MMSE, mini‐mental state examination; MP‐RAGE, 3D magnetization prepared rapid gradient echo.

a Not included in the matching.

### 2.2 Imaging acquisition and preprocessing

Participants had undergone a standardized protocol for high‐resolution MRI T1 scans of the brain as previously described (Clifford R. Jack et al., 2008). T1 imaging acquisition parameters were: TR = 2,400 ms, minimum full TE, TI = 1,000 ms, flip angle = 8°, 24 cm field of view, acquisition matrix of 192 × 192 × 166 and with 1.25 × 1.25 × 1.2 mm3 slice size. We used CAT12 (Computational Anatomy Toolbox) (Gaser & Dahnke, 2016) and SPM12 (Statistical Parametric Mapping,www.fil.ac.uk/spm) to perform voxel‐based morphometry (VBM). This included correcting the bias‐field distortions and noise removal, skull stripping, normalization to standard space and brain tissue segmentation into gray matter, white matter, and cerebrospinal fluid. Gray matter segments were modulated to represent actual gray matter volume. We then performed a biologically informed compression of the VBM data to the 673 gray matter parcels based existing in‐vivo brain parcellation (600 cortical gray‐matter parcels from Schäfer, 36 subcortical gray‐matter parcels from Brainnetome, and 37 cerebellar parcels from Buckner (Fan et al., 2016; Schaefer et al., 2018; Yeo et al., 2011). Thus, gray matter volume of each participant was represented by 673 features, each representing an individual parcel volume of that subject. The following analyses were performed on this data.

### 2.3 Statistical analysis of gray matter volume

Statistical analysis of gray matter volume of 673 parcels included three consecutive parts, suggested by Bludau and colleagues (Bludau et al., 2018); generating reference statistics, permuted statistics, and a family‐wise error (FWE) correction for multiple comparisons. Here we used an n‐way analysis of variance (ANOVA), to test the effect of age, cognitive status (HC, MCI, AD), SDB status, and SDB‐by‐cognitive status interaction, separately as independent variables (factors), on gray matter volume of each parcel as the dependent variable. The *F* values (per parcel) of this ANOVA were considered as the reference statistics. In the subsequent permutation statistics for each factor, we randomly shuffled the labels for that factor 10,000 times, replicated the analysis and recorded the *F* values to build a null‐distribution. The comparison of the reference statistic with this distribution then allows nonparametric inference per parcel and factor, yielding uncorrected *p*‐values. Importantly, however, we also recorded, per replication of the permutation, the highest statistics in the random data across the entire set (i.e., 673 brain regions), building a null‐distribution for FWE correction. The threshold corresponding to *P*_FWE_ < .05 was then provided by the (set‐wise maximum) value exceeded only in 5% of the replications.

### 2.4 Age prediction

**Figure 1:**
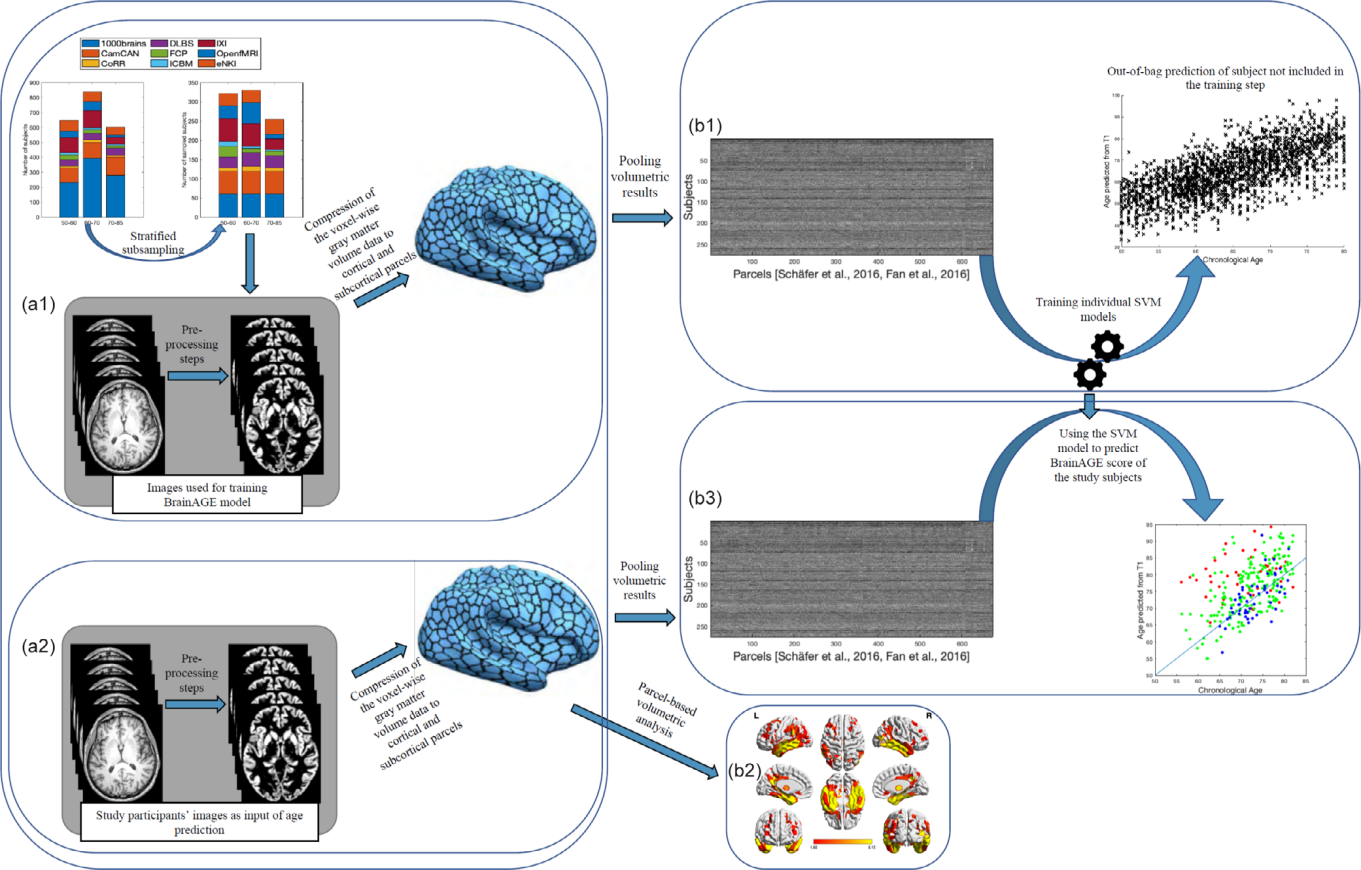
Main processing steps for parcel‐based volumetric study and age prediction based on gray matter morphometry. (a1) T1 brain images of 2089 non‐demented age, sex, and site stratified subjects were acquired through several imaging databases for the development of the age‐prediction model (training images). To obtain voxel‐based gray matter volume data, standard pre‐processing steps including normalization, segmentation, and modulation for nonlinear transformations have been done using Computational Anatomical Toolbox 12 (CAT12). A biologically informed compression of the voxel‐wise gray matter volume data to 600 cortical and 73 subcortical regions was applied accordingly. (b1) Parcel‐based representations of individual neuroanatomy were then used as input for training the support vector machine (SVM) used for the age‐prediction model. (a2) Similar pre‐processing steps were done on T1 brain images of study‐specific participants with and without sleep‐disordered breathing (study‐specific images). Parcel‐based results were used in two parallel analyses; (1) (b2) inputted to partial ANOVA tests for gray matter volume assessment according to the presence of sleep‐disordered breathing and cognitive status as contrasts and (2) (b3) inputted in the age prediction SVM model developed on the training images. ANOVA, analysis of variance

Brain age was estimated from the atlas‐based representations of individual brain anatomy using a support vector machine (SVM) ensemble model. An independent (reference) large dataset consisting of 2089 (Figure 1a1) subjects (between 55 and 85 years old) was compiled from several large public and private datasets including 1000Brains (Caspers et al., 2014), Cambridge Centre for Aging and Neuroscience or Cam‐CAN (Shafto et al., 2014), OpenfMRI (Poldrack et al., 2013), Dallas Lifespan Brain Study or DLBS, Consortium for Reliability and Reproducibility or CoRR (Zuo et al., 2014), IXI, and Enhanced Nathan Kline Institute‐Rockland Sample or eNKI‐RS (Nooner et al., 2012). Given the imbalance between age brackets, sites, and sex, we performed a stratified subsampling, choosing the same number of men and women, as well as similar numbers across age‐brackets and a maximum of 30 subjects per age‐bracket and sex per site. The actual subjects sampled in each replication from the overall database were drawn from the pool independently at random without replacement. Each of these sampled sets was then used to fit an individual SVM providing a weak learner for the ensemble which was applied to the test data, that is, the ADNI sample. The process was repeated 10,000 times, yielding 10,000 age predictions based on models trained on (different) balanced subsamples of the multi‐cohort reference data. These predictions were then averaged (“bagging”) to yield the final age prediction based on the 673‐parcel representation of the voxel‐based morphometry data (Becker, Mahlke, Reckert, Eickhoff, & Ritz‐Timme, 2019). Each subject's BrainAGE score was finally calculated as bagged predicted age minus chronological age for each subject (Figure 1).

### 2.5 Data availability

The original data used in this manuscript are publicly available in the online address of the ADNI database at http://adni.loni.usc.edu/data-samples/access-data/.

## 3 RESULTS

Each group with SDB and without SDB were comprised of 24 AD, 111 MCI, and 30 HC participants. There was no statistically significant difference in demographic variables, cognitive status, and presence of the APOE4 allele between SDB groups. Table 1 summarizes the characteristics of all study groups.

### 3.1 Effects on gray matter volume

**Figure 2:**
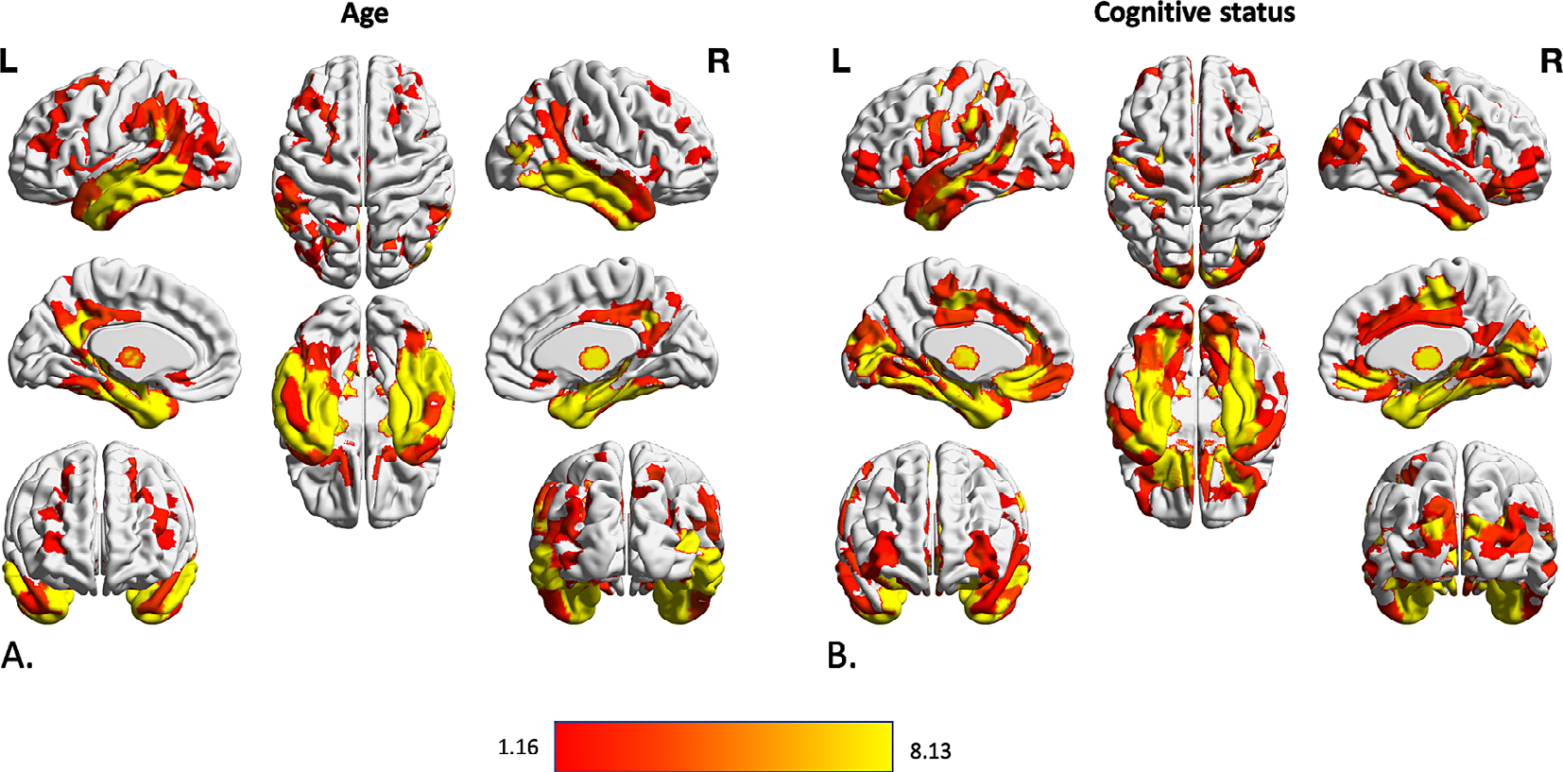
Association between volumetric data of cortical and subcortical parcels and age and cognitive status of subjects. Gray matter volume differences in 600 cortical parcels and 73 subcortical volume was assessed using three steps of using *F* value of an n‐way analysis of variance as reference statistics, running 10,000 permutations per randomly shuffling different parcels, under the assumption of label exchangeability, and correction of *p* values using family‐wise error (FWE) method. Significant parcels are illustrated as the heated areas on the brain maps considering (a) age and (b) cognitive status. Since there were no significant results regarding SDB presence or SDB‐by‐diagnosis interaction, results according to these factors have not been illustrated here. SDB, sleep‐disordered breathing

There were strong (*P*_FWE_ < .001) and widespread negative associations of regional gray matter volume with “age,” in particular in the bilateral temporal lobes, bilateral prefrontal, middle and superior frontal areas, bilateral medial and lateral occipital areas, cerebellum and thalamus, caudate and putamen in the subcortical gray matter (Figure 2a). The “cognitive status” was significantly associated with reduced gray matter volume in many bilateral parcels with dominancy in the left hemisphere (*P*_FWE_ < .001). Bilateral temporal lobes including fusiform gyri, medial temporal lobes, and hippocampal formations, and inferior and middle temporal lobes, as well as bilateral insula, middle frontal, and cingulate cortices, as well as left superior frontal cortex had significantly lower volume in participants with MCI and particularly AD (Figure 2b). In turn, when testing for effects of SDB status and SDB‐by‐cognitive status interaction, we found no significant region anywhere in the brain (all *P*_FWE_ > .05).

### 3.2 Effects on estimated brain age

**Figure 3:**
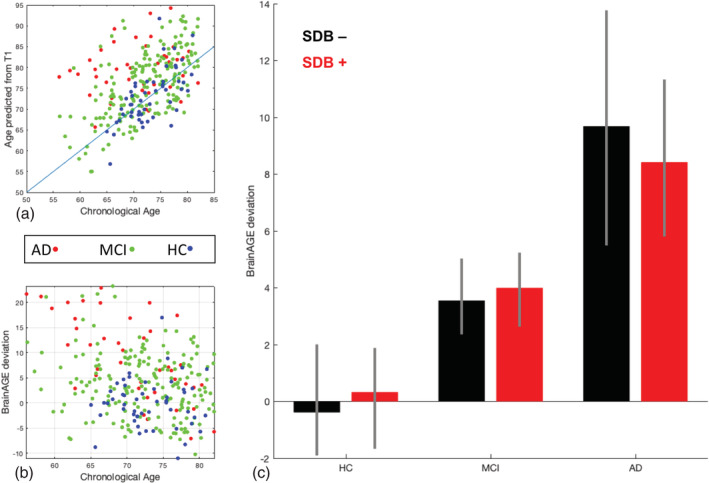
Results of the BrainAGE prediction method based on the presence of sleep‐disordered breathing and cognitive status. (a) Relationship between chronological age and the predicted age from T1 images in Alzheimer's disease, mild cognitive impairment, and healthy control groups. There is an evident higher predicted age for the participants with Alzheimer's disease and mild cognitive impairment compared to the healthy control group, in accordance with advanced pathological brain aging in the Alzheimer's disease course. (b) The BrainAGE score shows positive and bigger deviation from chronological age in Alzheimer's disease and mild cognitive impairment groups. (c) Despite the significantly higher BrainAGE deviation associated with Alzheimer's disease and mild cognitive impairment, no significant deviation was seen between the BrainAGE score of sleep‐disordered breathing subgroups

The mean absolute error (MAE) between predicted and chronological age in the HC group was 3.59 years, indicative of the very good performance of the ensemble prediction model. We then calculated the BrainAGE score as the per‐subject difference between predicted and chronological age and tested for its association with cognitive status, SDB status, and the SDB‐by‐cognitive status interaction. Participants with MCI and in particular AD showed an advanced brain age (on average 4.0 (95% confidence interval or CI: 2.6–5.4) and 9.1 (95%CI: 5.8–12.4) years, respectively) (Figure 3), in line with previous studies. However, there was no significant effect on BrainAGE scores associated with SDB status, nor was there a positive SDB‐by‐cognitive status interaction, suggesting that SDB may not lead to advanced brain aging (Figure 3c).

## 4 DISCUSSION

Our findings confirmed previously reported gray matter atrophy and accelerated biological brain aging in patients with MCI and AD, corroborating the robustness and validity of our analytical approach. However, importantly, we were not able to demonstrate any effect of SDB, independently or in interaction with cognitive status, on either regional gray matter volume or brain aging score. Of note sample sizes of subjects with SDB in the HC and AD groups were small. To our knowledge, the current sample represents the largest neuroimaging study in SDB, which is actually a by‐product of a large, openly shared dataset aimed primarily at a different purpose, namely the investigation of AD and dementia. This not only highlights the potential of shared data with broader phenotypical information, but also provides important future perspectives arising from the increased availability of such datasets. In this context, we would like to particularly highlight the UK Biobank, which through its extensive medical and social history taking will likely have a profound impact on the investigation of the neurobiological effects of various common medical conditions (Sudlow et al., 2015). Given the sheer size of the dataset and the rather restricted age‐range, there will be hundreds of cases, as well as potentially thousands of control subjects for medical conditions like SDB, easily outnumbering any individual, monocentric study (Campos et al., 2020; H. Wang et al., 2019). Thus, a (ideally pre‐registered) replication of the current analysis in that large dataset seems warranted in future. As another possible limitation here, the groups were heterogeneous in terms of clinical characteristics and imaging specifications. We used propensity‐score matching and stratified subsampling of external datasets to minimize the effects of heterogeneity. As previously mentioned on publications using the ADNI database (Bubu et al., 2019; Osorio et al., 2015), the self‐reported measure of SDB can be influenced by both the recall bias of cognitively impaired subjects, as well as by a high prevalence of undiagnosed OSA in the general population, therefore increasing the probability of false‐negative cases considering SDB diagnosis (Osorio et al., 2015). Moreover, assessment of SDB severity and disease duration were not available in the ADNI data.

### 4.1 Gray matter volume alterations in AD and SDB

One of the main characteristics of MCI and AD is generalized gray matter loss in the brain, which mostly starts in the medial temporal lobe and multimodal association areas (Fox & Schott, 2004; C. R. Jack et al., 2004; Karas et al., 2004; Pasquini et al., 2019). Neuroimaging meta‐analyses in AD have demonstrated atrophy in the medial temporal lobe, limbic regions (left parahippocampal gyrus, left posterior cingulate gyrus, amygdala, and uncus), thalamus, temporal, parietal, frontal and cingulate cortices (W.‐Y. Wang et al., 2015; Yang et al., 2012). A similar, but milder distribution of gray matter atrophy is evident in the brain of patients with MCI (Nickl‐Jockschat et al., 2012; Yang et al., 2012). In accordance with the previous brain volumetric studies, we found diffuse gray matter loss in MCI and AD. The atrophy was mainly located in the bilateral temporal lobe and medial temporal areas with higher intensity in AD compared to MCI.

Assessing the volumetric changes due to SDB, we did not observe any significant alteration in gray matter volume, neither in HC subjects, nor in patients with MCI or AD. Furthermore, self‐reported SDB interaction with cognitive status (HC, MCI, AD) revealed no associations with gray matter volume. Historically, there has been an inability to replicate results among the brain imaging studies of SDB in non‐demented populations. While several studies have reported gray matter atrophy in the insula, amygdala, middle and lateral temporal regions, and cerebellum in non‐demented populations with SDB (Joo et al., 2013, 2010; Morrell et al., 2010; Torelli et al., 2011; Weng et al., 2014), others have either shown no associations (O'Donoghue et al., 2005; Yun et al., 2017) or even enhancement in the gray matter volume in the motor cortices, prefrontal cortex, thalamus, putamen, and the hippocampus (Baril et al., 2017; Kumar et al., 2014; Lin et al., 2016; Lundblad et al., 2014; Rosenzweig et al., 2013; Taylor et al., 2018). In addition, there is a general lack of longitudinal studies, which would enable the study of nonlinear associations between SDB and cortical atrophy, suggested by the present cross‐sectional findings. Despite these important gaps in the literature, three neuroimaging meta‐analyses have demonstrated that OSA is associated with gray matter atrophy in a few selected regions including the amygdala and hippocampus (Tahmasian et al., 2016), as well as cingulate, right central insula, right middle temporal gyrus, right premotor cortex, and cerebellum (Shi et al., 2017; Weng et al., 2014).

The observed null association between SDB and gray matter volume should, however, be interpreted with caution. Firstly, it has been suggested that aging may have partially protective mechanisms against SDB, such as reduced production of oxidative stress after apneas and decreased blood pressure and heart rate responses after arousals (Baril et al., 2017). The average old age of ADNI subjects (~75 years‐old) could, therefore, explain this nonsignificant association between SDB and brain morphometry. Despite numerous individual studies and meta‐analyses focused on the changes in gray matter in middle‐aged patients with OSA, there are few studies on gray matter changes in older adults with SDB and, to our knowledge, neither have found decreases in thickness or volume in cortical gray matter (Sébastien Celle et al., 2009; Cross et al., 2018; Lutsey et al., 2016). Secondly, it is possible that SDB impairs selective brain functions (Canessa et al., 2018) or amyloid burden (Yun et al., 2017) before gray matter volume. Furthermore, differential diagnosis between *SDB‐related* and *age‐related* brain atrophy is difficult in single‐point observational studies, particularly in those cases in which groups are matched by age and cognitive status. Thirdly, this could also be a sign of a) survival bias, as most patients with SDB may have transitioned to AD and only those with very low cortical atrophy or high in cognitive reserve at disease onset would remain as HC or MCI at cross‐section; or b) selection bias due to matching by the APOE4 allele, as it has been reported that the APOE4 allele interacts with brain aging scores measured by the BrainAGE method, revealing potential neuronal compensation in healthy APOE4+ adults (Scheller et al., 2018), which could also result in null findings. Fourthly, we did not account for other comorbidities and possible confounders alongside age or presence of the APOE4 allele in the prediction models (Gozal, 2018). Finally, previous MRI studies mostly recruited patients with polysomnography‐diagnosed OSA from sleep clinics, which might be a different population from those recruited in memory clinics with a self‐reported assessment of SDB based on their clinical interview.

Interestingly, we were not able to demonstrate any interaction between SDB and MCI or AD with brain atrophy. This is indicative that despite the frequent clinical co‐occurrence of SDB and AD, there may be no synergy between them in accelerating gray matter atrophy. Recent investigations using cerebrospinal fluid and PET imaging suggest an interplay between amyloid production/clearance and SDB (Bubu et al., 2019; Liguori et al., 2017; Sharma et al., 2018; Spira et al., 2013; Yun et al., 2017). These include an impairment in the cerebrospinal fluid–interstitial fluid exchange (Ju et al., 2016), cerebral edema secondary to an intermittent hypoxia (Spira et al., 2018) (similar to the increase in brain volume and pseudoatrophy observed in multiple sclerosis), and compensatory excessive neuronal synaptic activity (Polsek et al., 2018) in SDB, all of which could potentially lead to an increase in beta‐amyloid deposition and its clearance reduction. It is, therefore, possible that the presence of SDB is associated with AD risk only through beta‐amyloid deposition (Bubu et al., 2019; Sharma et al., 2018) or altered brain function (Chen et al., 2018; Park et al., 2016; Thomas, Rosen, Stern, Weiss, & Kwong, 2005), as mentioned before. While amyloid burden has been linked to SDB in several observational studies (Bubu et al., 2019; GBD 2016 Disease and Injury Incidence and Prevalence Collaborators et al., 2017; Sharma et al., 2018), a recent study on non‐demented elderly subjects, has found no association between self‐reported sleep disturbances and brain amyloid PET burden (Gabelle et al., 2019). In addition, in our results, we expected an interaction with MCI or AD where it is generally accepted that neuronal loss follows amyloid deposition. More studies are needed to better understand the compensatory increase in gray matter volume in SDB suggested by several studies, as well as the precise progression of brain atrophy in AD, as both may have contributed to obtaining such negative findings.

### 4.2 BrainAGE prediction in AD and SDB

Brain age prediction methods have been previously used in cognitively normal subjects (Aycheh et al., 2018; Franke et al., 2012). In addition, several studies have used the ADNI dataset and other datasets of middle‐aged adult and elderly population with MAE ranging from 3.8 to 6 years (Cole et al., 2019; Varikuti et al., 2018). Conversely, in our study, an advanced sensitive BrainAGE estimation method has been implemented to detect pathologic brain aging. A repeated support vector machine (SVM) models were fitted on parcel‐wise gray matter volume data of on stratified subsamples from external cohorts, making the model notably less sensitive to heterogeneity in images (Varikuti et al., 2018). In addition, compared to previous studies on the middle‐aged adults and elderly subjects (Cole et al., 2019), while using multiple datasets for training prediction model, our age prediction results were accurate with an MAE of 3.6 years in older adults and elderly subjects. Replication of previous findings in patients with AD, taken together with acceptable MAE, is indicative of the reliability of our proposed method in gray matter volume assessment and age estimation.

While there is no exact definition for accelerated brain aging, the BrainAGE score has been shown to be a sensitive predictor of disease progression in dementia (Cole et al., 2019; Gaser et al., 2013; Löwe et al., 2016). Previous findings on increased BrainAGE score in MCI and AD course (Beheshti, Maikusa, & Matsuda, 2018; Caballero, Klöppel, Dichgans, & Ewers, 2016; Liem et al., 2017), are in agreement with the reported accelerated aging of the demented brain shown in‐vivo and ex‐vivo studies (Mecocci et al., 2018). The BrainAGE score in studies using ADNI ranged from almost zero for patients with stable MCI, to 5.7–6.2 years for patients with progressive MCI, and reached up to 10 years for patients with AD (Cole et al., 2019). We found the average 4.1 and 9 BrainAGE scores in patients with AD and MCI, in agreement with previous findings using ADNI data. Since we did not distinguish patients with progressive from stable MCI, our results in the MCI group were modest compared to other studies including patients with late or progressive MCI.

## 5 CONCLUSIONS

The association between sleep, gray matter volume and cognitive functions has been demonstrated previously (Tahmasian et al., 2019; Takeuchi et al., 2018). Here, we have demonstrated the acceleration of brain atrophy and advanced brain aging in MCI and AD participants from the ADNI cohort compared to HCs. We further found that self‐reported SDB in subjects with a diagnosis of HC, MCI or AD was neither associated with gray matter volume reduction, nor with accelerated brain aging. While SDB is suggested to propagate the aging process, amyloid burden and cognitive decline to AD, it may not necessarily associate with brain atrophy and the estimated brain age in AD progression. Reproducibility of neuroimaging findings is one of the major issues in science. While every effort has been done to increase the robustness and validity of our findings, future analyses will inevitably benefit from inclusion of well characterized and objectively diagnosed SDB phenotypes, ideally collected from studies that were specifically designed to investigate AD‐SDB interaction.

## CONFLICT OF INTEREST

The authors declare no potential conflict of interest.

## Data Availability

Data used in the preparation of this article were obtained from the Alzheimer's Disease Neuroimaging Initiative (ADNI) database (adni.loni.usc.edu). As such, the investigators within the ADNI contributed to the design and implementation of ADNI and/or provided data but did not participate in analysis or writing of this report. A complete listing of ADNI investigators can be found at: http://adni.loni.usc.edu/wp-content/uploads/how_to_apply/ADNI_Acknowledgement_List.pdf.
